# Dueling capsules: novel devices for GI bleeding diagnostics

**DOI:** 10.1016/j.igie.2024.04.018

**Published:** 2024-06-25

**Authors:** Shuji Mitsuhashi, Andrew C. Storm

**Affiliations:** Division of Gastroenterology and Hepatology, Mayo Clinic School of Medicine, Rochester, Minnesota, USA

Today’s advancements in medical technology have introduced a new era of diagnostics, particularly in the field of GI health. Among these innovations, the introduction of blood sensor capsules has emerged as groundbreaking solutions for detecting blood within the GI tract. Importantly, GI bleeding is the most common admission for GI diagnosis to the inpatient hospital. Two recent case reports and series highlighted the potential of these capsules: 1 using PillSense (EnteraSense, Galway, Ireland) and the other HemoPill (Ovesco, Tübingen, Germany). Both are single-use, ingestible capsules containing sensors that detect blood by analyzing the absorption of multiple wavelengths of light.

As outlined in a case series by Szarca et al,^1^ PillSense, a blood-detecting capsule cleared by the U.S. Food and Drug Administration, has demonstrated remarkable accuracy in detecting even minute traces of blood in the upper GI tract in 1 case, enabling early intervention with EGD and life-saving treatment. PillSense was also reported to have safely ruled out active or recent GI hemorrhage for a patient with questionable GI bleeding with a Glasgow-Blatchford score of 2, preventing inpatient admission and successfully facilitating upper endoscopy in an outpatient setting, optimizing hospital resources. Notably, a recent prospective clinical trial from the Mayo Clinic reported the PillSense device’s sensitivity and specificity for upper GI bleeding to be 92.9% (95% confidence interval, 76.5-99.1; *P* = .02) and 90.6% (95% confidence interval, 82.9-95.6; *P* < .001), respectively.^2^ Importantly, no adverse events or deaths associated with the PillSense system were reported in the Mayo Clinic study or the aforementioned cases.

Another capsule, not yet marketed or approved in the United States, was outlined by a case report by Rainer et al.^3^ In this case report, HemoPill demonstrated effectiveness in detecting bleeding in the small bowel in a patient with multiple comorbidities and was reportedly used to successfully guide the direction of endoscopic approach to treat suspected GI bleeding. In this case, HemoPill was used to confirm bleeding from a previously identified jejunal lesion during video capsule endoscopy (VCE) that had been performed 2 months previously, without having to repeat another lengthy and costly VCE procedure.

Unlike the PillSense device, the sensitivity and specificity of HemoPill are not well documented in a prospective trial of suspected upper GI bleeding. Nonetheless, in preclinical studies in a porcine model, HemoPill demonstrated sensitivity and specificity of the sensor in upper GI bleeding of more than 80%.^4^ Notably, HemoPill exhibited no adverse events in this study or the referenced case report.^4^^,^^5^

Neither capsule device has been thoroughly investigated for the ability to detect bleeding in the small bowel. Primarily, both types have been studied in cases of upper GI bleeding, with confirmation through EGD, encompassing the esophagus, stomach, and distal duodenum. Despite this limited scope of research, the use of these blood sensor capsules holds promise not only in the triage and decision-making around upper GI bleeding but potentially also in addressing small-bowel bleeding, as suggested by findings in Rainer et al.

In terms of diagnosing small-bowel bleeding, VCE has been predominantly used over the years to guide endoscopists to locate bleeding and determine which directionality and endoscopic approach to use.^6^ However, given the complexity and expertise needed to deploy, record, and interpret VCE, it is important to note that not all healthcare facilities may have the necessary resources or trained personnel to perform VCE. When VCE is performed, the collected video footage must be reviewed and interpreted by a trained gastroenterologist. Because of the large volume of video data generated during the procedure, the interpretation process can be time-consuming, potentially resulting in delays in scheduling a procedure and longer wait times for patients.^7^ In contrast, optical blood-sensing capsules do not require cutaneous abdominal sensors for patients as does VCE and only require a receiver to be held within proximity of the patients for the duration of the procedure. The device can be easily dispensed and results interpreted by trained nonphysician personnel, simplifying the process.^2^ Interestingly, the case report by Raina et al demonstrated efficacy for blood detection in small-bowel bleeding that was previously believed to require VCE, which is promising for the future.

In conclusion, 2 new *iGIE* reports demonstrate the effectiveness and safety of novel, minimally invasive capsules in managing luminal bleeding. Although the PillSense device is approved, available in the United States, and appears to have the highest sensitivity and specificity for detection of upper GI bleeding, no head-to-head data are available. The simplicity and efficiency of blood-sensing capsules present a compelling solution for diagnosing both upper GI and potentially small-bowel bleeding. Although blood-sensing capsules are not positioned to replace all VCE, in select cases where a yes or no answer to the question “Is there active bleeding in the GI tract?” is needed, they may aid in triaging patients, especially in facilities without VCE capabilities. Other potential use cases include monitoring of patients with high risk of developing recurrent bleeding after an endoscopic therapy and to assist in determining the need for a bowel preparation when upper versus lower GI bleeding is in question. The clear potential for cost-effectiveness and streamlined patient care underscores their value in a busy, modern GI practice. However, rigorous clinical evaluation and real-world implementation are essential to fully understand these devices’ performance characteristics and comparative advantages over traditional methods. Nonetheless, blood sensor capsules represent a promising step forward in GI diagnostics, offering hope for improved patient outcomes and more accessible healthcare solutions.

## Disclosure

The following author disclosed financial relationships: A. C. Storm: Research support from Apollo Endosurgery, Boston Scientific, Endogenex, Endo-TAGSS, Enterasense, MGI Medical, OnePass, and SofTac; consultant for Ambu, Boston Scientific, Envision Endoscopy, Intuitive, Medtronic, Microtech, and Olympus. All other authors disclosed no financial relationships.
